# Rectal mucosal inflammation, microbiome, and wound healing in men who have sex with men who engage in receptive anal intercourse

**DOI:** 10.1038/s41598-024-80074-1

**Published:** 2024-12-30

**Authors:** Vanessa E. Van Doren, Cassie G. Ackerley, Robert A. Arthur, Phillip M. Murray, S. Abigail Smith, Yi-Juan Hu, Colleen F. Kelley

**Affiliations:** 1 The Hope Clinic of the Emory Vaccine Center, Division of Infectious Diseases, Department of Medicine, Emory University School of Medicine, 500 Irvin Court #200, 30030 Decatur, Georgia, United States; 2 Emory Integrated Computational Core, Emory University, Woodruff Memorial Research Building, Suite 7110, 101 Woodruff Circle, 30322 Atlanta, Georgia, United States; 3 Department of Biostatistics and Bioinformatics, Rollins School of Public Health, Emory University, 1518 Clifton Road, 30322 Atlanta, Georgia, United States; 4 Grady Health System, 80 Jesse Hill Jr Drive, 30303 Atlanta, Georgia, United States

**Keywords:** Mucosal immunology, Microbiome, Mucosal healing, Bacterial guilds, Receptive anal intercourse, HIV infections, Microbiome, Cytokines, Computational biology and bioinformatics

## Abstract

Mucosal injury is common during consensual intercourse and induces an inflammatory response that could contribute to pathogen transmission including HIV. Here, we compared mucosal immune and microbiome responses to experimentally induced mucosal injury between men who have sex with men engaging in receptive anal intercourse (MSM-RAI) and men who do not engage in RAI (controls), all without HIV. Rectal mucosal secretions were collected from adult MSM-RAI (*n* = 19) and controls (*n* = 6) via anoscopy before and up to eight days after experimentally induced injury. Mucosal healing was evaluated by repeated injury surface area measurements with digital imaging. MSM-RAI demonstrated overall significantly higher concentrations of pro-inflammatory cytokines and a distinct rectal microbiome compared with controls. Wound healing was numerically faster in MSM-RAI but did not meet statistical significance (*p* = 0.09). Different cytokine injury response patterns were observed between MSM-RAI and controls; however, IL-6 and IP-10 were important mediators in both groups. Microbial guilds, particularly from the *Lachnospiraceae* and *Prevotellaceae* families, were associated with rectal mucosal inflammation. This work is the first experimental study of rectal mucosal injury and the immune environment in healthy humans and provides a more nuanced understanding of rectal mucosal inflammation after injury, which can inform our understanding of HIV transmission.

**Supplementary Information:**

The online version contains supplementary material available at 10.1038/s41598-024-80074-1.

## Introduction

Gay, bisexual, and other men who have sex with men (MSM) accounted for 65% of new HIV infections in the United States in 2022^1^. One potential contributor to this rate is the efficiency of HIV transmission across the rectal mucosa (RM), where approximately 70% of infections are thought to occur among MSM^2^, as compared to penile transmission^3^. To design effective prevention measures to reduce HIV transmission, it is important to understand the biologic factors that could influence the integrity of the RM barrier.

While few participants sustain visible injury after consensual sex, subclinical mucosal injury can be frequently identified by UV light in the vagina^4,5^. The RM is composed of a single layer of columnar epithelium and is potentially more susceptible to microinjury and permissive to HIV transmission than vaginal stratified squamous epithelium^3^. Interactions between mucosal immune and epithelial cells promote rapid repair of the epithelial barrier after injury; small RM wounds have been shown to heal in 8–12 days in humans^6^. Important mediators of the immune response to injury include the production of pro-inflammatory cytokines, including TNF-α, IL-8, and IL-1β^7^. Mouse models have highlighted the importance of inflammatory cascades, including the IL-1 superfamily, in gut mucosal wound healing^8–10^. In inflammatory bowel disease models, gut mucosal IL-1β and IL-8 secretion has also been found to predict relapse^11^, and sustained mucosal IL-17A exposure has been shown to inhibit inflammation resolution^12^. Therefore, mucosal injury during intercourse could increase HIV transmission risk via mucosal barrier disruption, increased pro-inflammatory cytokine production and HIV target cell availability, and potentially by providing a hematogenous route for rapid systemic HIV spread.

RM inflammation and the microbiome composition likely impact HIV acquisition risk, as demonstrated for penile^13^ and vaginal^14,15^ HIV transmission. Prior to HIV seroconversion, distinct baseline rectal microbiome differences and increased baseline serum pro-inflammatory cytokines have been found in MSM participants who later acquired HIV compared to matched groups that did not^16,17^, but more research is needed to identify significant associations between the RM microbiome and inflammation and how behaviors like receptive anal intercourse (RAI) affect HIV transmission. Compared to men who do not engage in RAI, we previously found that MSM who engaged in RAI demonstrated a unique RM immune environment characterized by a *Prevotella-*enriched rectal microbiota, increased percentage of ex vivo stimulated rectal T cells producing inflammatory cytokines, and increased rectal expression of genes involved in mucosal injury and repair^18^. We also found that rectal application of hyperosmolar lubricant, which has been shown to induce epithelial cell damage and cytotoxicity, was associated with a rectal microbiome composition shift towards increased *Prevotella* and decreased *Bacteroides* relative abundance among MSM engaging in RAI^19^. It was previously demonstrated in mice that IL-6 is essential for post-injury intestinal regeneration, and its absence is associated with gut microbial dysbiosis and significantly reduced diversity^20,21^. Together, these studies suggest that gut microbiome composition may affect or be affected by gut mucosal injury and inflammation.

Given the role that mucosal injury could play in facilitating HIV transmission and the known differences in the RM immune environment among MSM engaging in RAI, we sought to compare the RM immune and microbiome response to experimentally induced mucosal injury between MSM who engage in RAI (MSM-RAI) and men who do not engage in RAI (controls). To do this, we adapted an experimental mucosal injury animal model to humans and measured changes in RM inflammatory cytokines, the microbiome, and wound healing after mucosal injury. We hypothesized that the RM among MSM-RAI, as compared to controls, would exhibit a distinct inflammatory response and unique microbiome perturbations after mucosal injury.

## Methods

### The clinical cohort

Men aged 18–59 years without HIV in good health were recruited in Atlanta, Georgia and enrolled between March 2020-April 2022. Nineteen MSM who had engaged in receptive anal intercourse at least three times in the past month (MSM-RAI) and six men who reported no sex with men and had never engaged in RAI (controls) were enrolled. Exclusion criteria included a history of inflammatory bowel disease or other significant gastrointestinal tract disturbance, bleeding disorder, systemic immunomodulatory agent or exogenous hormone use, antibiotic use in the four weeks prior to biopsy, and unwillingness to abstain from RAI from three days before biopsy through the study duration.

The study consisted of five visits. During the screening visit, eligible participants provided informed consent and then underwent a brief sexual and medical history, physical examination, rapid HIV testing, and blood collection. At the baseline study visit (day 0), a disposable self-illuminating proctoscope with camera attachment (THD ProctoStation: https://www.thdlab.com/healthcare-professionals/products/thd-proctostation) was placed in the rectum, and baseline RM secretions were collected with polyvinyl acetal sponges pre-wet with 35 ul sterile PBS for cytokine analyses and polyurethane swabs for microbiome analyses. Methylene blue was applied to the RM surface to facilitate injury site identification at subsequent visits. Approximately 6 cm from the anal verge, two experimental RM injuries were then made using a biopsy forceps (Olympus EndoJaw Disposable Biopsy Forceps, 3.3 mm diameter: https://medical.olympusamerica.com/products/endojaw-disposable-biopsy-forceps). Baseline wound images were then collected using the proctoscope camera. Participants returned on days 2, 5, and 8 (+/- 1 day each). At each visit, the proctoscope with camera attachment was placed again, and follow-up images were collected to assess wound healing. RM secretions for cytokine and microbiome analyses were also collected directly adjacent to the injury sites at follow-up visits. When the injury site was unable to be identified, rectal secretions were collected as close to the presumed injury location as possible. The Institutional Review Board (IRB) at Emory University approved this study (IRB00117533), all methods were performed in accordance with the relevant guidelines and regulations, and informed consent was obtained from all participants in accordance with the Declaration of Helsinki.

### Cytokine quantification

Twelve RM cytokine concentrations (IP-10, IL-1β, TNF-α, MCP-1, IL-17A, IL-6, IFN-γ, IL-12p70, IL-8, IL-4, IL-10, TGF-β1) were measured at baseline (day 0) and after experimental RM injury (days 2, 5, and 8). Rectal secretions collected at each visit with polyvinyl acetal sponges were stored at − 80 °C. A total of 98 samples were analyzed from 25 participants over four visits (two samples were mislabeled and discarded). One sponge per participant was subsequently thawed, and cytokines were extracted using the Sigma Luminex cytokine extraction protocol for cervical, vaginal, and saliva secretions (https://www.sigmaaldrich.com/deepweb/assets/sigmaaldrich/marketing/global/documents/939/349/br1088en_ms.pdf). Cytokine concentrations (pg/mL) were then measured using the LegendPlex Multi-Analyte Flow Assay Kit: Human Essential Immune Panel per manufacturer instructions (see Supplemental Methods for cytokine extraction and quantification details).

#### Inflammation score calculation

Nine cytokines (IP-10, IL-1β, TNF-α, MCP-1, IL-17A, IL-6, IFN-γ, IL-12p70, IL-8) were designated as primarily pro-inflammatory and three (IL-4, IL-10, TGF-β1) as primarily anti-inflammatory^22^. Using previously published scoring criteria^14,15^, a composite inflammation score incorporating all 12 cytokines (12C IS) was calculated for each participant at each visit (see Supplemental Methods for scoring criteria details).

#### Cytokine statistical analyses

Cytokine concentrations and 12C IS inflammation scores were compared between the MSM-RAI and control groups overall (i.e., as trajectories across time points) and at each of the four study visits, as well as changes between baseline and days 2, 5, and 8, using discovery-based linear decomposition modeling (LDM), a statistical method which we have previously described (see Supplemental Methods for details)^23^. We used LDM to provide a global p-value (with significance set at *p* < 0.05) to assess overall cytokine profile differences between groups and visits as well as individual cytokines that were significantly contributing to this global difference.

We detected consistent significant, biologically plausible global cytokine differences between the MSM-RAI and control groups and thus wanted to identify the individual cytokines that were the strongest contributors to this global difference. Given the novel, hypothesis-generating nature of this study, our small number of absolute discoveries (12 cytokines), and consistent with prior publications analyzing similar data^24^, we chose a nominal FDR level of 20% in order to avoid obscuring true discoveries. A very stringent FDR may be appropriate in the setting of a much larger number of absolute discoveries (e.g. thousands such as seen in transcriptomic studies), but the same FDR in the setting of a low number of discoveries could obscure important associations. We report unadjusted p-values in the manuscript text and have provided both FDRs and unadjusted p-values for all analyses as supplemental tables. Inspection of mean concentration graphs revealed apparent peaks in the controls for IL-1β and IL-8 (baseline to day 2) and IL-6 (baseline to day 5); Mann-Whitney tests were conducted comparing both MSM-RAI and control concentrations at these timepoints for these three cytokines.

### Wound healing assessments

Digital images of experimentally induced RM injuries were collected at baseline and follow-up visits (Supplemental Fig. 1). We were not able to collect an image from every study visit, as we were sometimes unable to locate the injury site at subsequent visits, participants occasionally missed visits, and the camera equipment was sometimes unavailable. We collected images at 77/100 visits (77%). For five participants (three MSM-RAI, two controls), we were unable to collect any images. For three participants (all MSM-RAI), we were unable to collect images at their final visit.

Wound surface area in pixels was measured using ImageJ (https://imagej.net/ij/). Three of the co-authors (VVD, CFK, and PM) independently measured wound surface area for each image. When surface area differed between measurers by > 10%, discrepancies were discussed, and measurements were independently repeated. All measurements were subsequently < 10% discordant. We then averaged our three individual surface area measurements to determine the final measurement for each data point.

Two RM injuries were induced per participant, however only measurements for one injury site per participant were included in final analyses. When high-quality images for both injury sites were available, the site with the most data points was used. For example, high-quality images were available for RM 09 at three visits for injury site A and at four visits for injury site B, thus only the four data points from injury site B were included. After this rigorous process, 57 surface area measurements remained. From these, we were able to calculate healing trajectories for 18 participants (14 MSM-RAI, 4 control).

#### Wound healing statistical analyses

Wound surface area over time was compared between the MSM-RAI and control groups using a linear mixed effects model.

### Microbiota sequencing

A RM swab was collected at each visit to measure microbiome composition at baseline and after experimental RM injury. It was stored at -80 °C and subsequently thawed. A total of 100 samples were analyzed from 25 participants over four visits. DNA was extracted, amplified, and sequenced using methods described previously (further details in Supplemental Methods)^23,25^.

#### Microbiome statistical analyses

Differences in alpha diversity, beta diversity, taxon relative abundance, and taxon presence-absence between the MSM-RAI and control groups were assessed as overall trajectories across all four visits and as changes between baseline and days 2, 5, and 8 using methods described previously^26,27^. Alpha diversity, or the richness and evenness of species in a given community, was measured using the Chao1 and Shannon indices and compared between the MSM-RAI and control groups with a Wilcoxon test. Beta diversity, or between-sample dissimilarity in community composition, was measured using the Bray-Curtis and Jaccard indices, tested using PERMANOVA, and visualized with Principal Coordinates Analysis (PCoA). This was calculated using an amplicon sequencing variant (ASV) table and with vegan software package as described previously^6,7^. Taxon relative abundance, which provides the microbiome percentage that is comprised of a specific organism, was analyzed by LDM, which generates a global p value and then assesses associations at individual taxa^8^. Taxon presence-absence, which assesses the probability that a taxon is present within a given sample^9^, was then conducted with LDM, which again generated a global p value and individual taxa associations. All analyses were conducted at the genus level (see Supplemental Methods for further details regarding presence-absence and LDM analyses). Consistent with our cytokine analyses, global significance for microbiome analyses was set at *p* < 0.05, and we chose a nominal FDR of 20% for the assessments of individual taxa given the small number of causal taxa.

Among all participants (i.e., both MSM-RAI and controls), LDM was used to assess associations between microbiome relative abundance and the 12C IS. In order to identify taxa across all taxonomic levels and driver taxa (i.e., the highest level of taxa below which there are dense association signals) that were primarily responsible for this association, we then conducted a BOUTH analysis, which is a bottom-up testing algorithm developed to test tree-structured hypotheses by starting at the lowest available phylogenetic tree level and proceeding upward through successively higher taxonomic groupings^28^. Next, we conducted a SparCC network analysis to identify bacterial guilds^29^, which are groups of genera with positively or negatively correlated relative abundance without regard to taxonomic positions. SparCC estimates Pearson’s correlation coefficients between taxa, which are between − 1 and 1, and addresses the subcompositional incoherence inherent in correlation analyses of compositional data by applying a log-ratio transformation^30,31^. Because the MSM-RAI and control microbiome compositions were significantly different, we conducted a stratified network analysis. Within this, we located nodes that were significant in both the relative abundance-12C IS LDM and BOUTH analysis. We then used a stringent correlation coefficient cutoff of > 0.7 or <-0.7 to visualize the taxa that were most correlated with these inflammation-associated nodes in both MSM-RAI and controls. We used Flourish network plots (https://flourish.studio/visualisations/network-charts/) to visualize guilds.

## Results

### The clinical cohort

Demographic and clinical characteristics are presented in Table 1.

**Table 1:** Demographic data. MSM-RAI = men who have sex with men engaging in receptive anal intercourse.

Characteristic	MSM-RAI (*n* = 19)	Control (*n* = 6)	*p*-value
Median age in years (range)	31 (21–54)	29 (23–46)	0.8
Race n (%)			0.04*
Black	9 (47.37%)	0 (0%)	
White	9 (47.37%)	4 (66.67%)	
Asian	1 (5.26%)	1 (16.67%)	
Declined to Answer	0 (0%)	1 (16.67%)	
PrEP use n (%)	9 (47.37%)	0 (0%)	0.06

### Rectal mucosal inflammation was higher in secretions collected at baseline among MSM engaging in RAI compared to controls

**Fig. 1:**
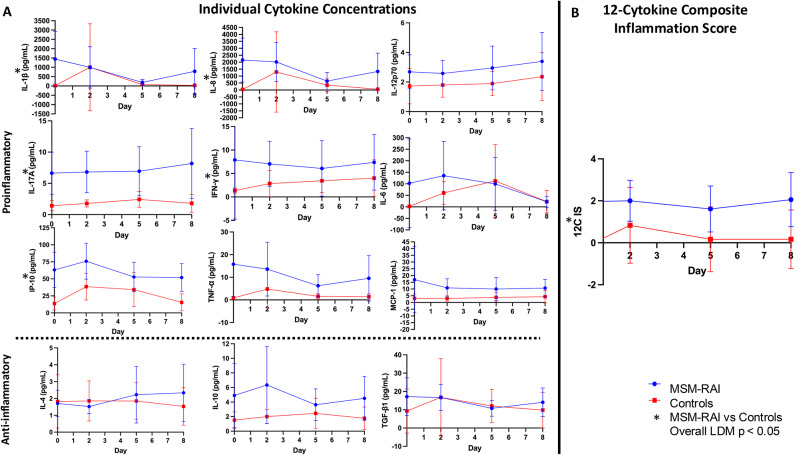
Rectal mucosal cytokine responses to experimentally induced injury in MSM engaging in RAI and controls. Twelve rectal mucosal cytokines were measured at baseline (day 0) and after experimental rectal mucosal injury (days 2, 5, and 8). Nine cytokines were classified as primarily proinflammatory (IL-1β, IL-17A, IP-10, IL-8, IFN-γ, TNF-α, IL-12p70, IL-6, MCP-1), and three cytokines were classified as primarily anti-inflammatory (IL-4, IL-10, TGF-β1). (**A**) Concentrations were compared between MSM who have frequent RAI (blue dots) and control men who have never had RAI (red squares) using LDM. (**B**) A 12-cytokine inflammation score (12C IS) was calculated incorporating the nine proinflammatory and three anti-inflammatory individual cytokines for each participant at each visit. Scores were again compared between MSM who have frequent RAI and controls using LDM. Error bars indicate 95% confidence intervals.

There was a marginally significant (*p* = 0.09) difference in overall cytokine profile across all study visits between the MSM-RAI and control groups with five significantly elevated cytokines in the MSM-RAI group (IL-1β, IL-17A, IP-10, IL-8, and IFN-γ; all *p* < 0.05 for individual cytokines, Fig. 1A and Supplementary Table 1). An LDM assessing MSM-RAI versus control differences at each visit demonstrated a global significant difference at day 0 (global *p* = 0.05, again with IL-1β, IL-17A, IP-10, IL-8, and IFN-γ significantly elevated in MSM-RAI; all *p* < 0.05 for individual cytokines, Supplementary Table 1) and a marginally significant difference at day 8 (global *p* = 0.09, with IL-1β, IL-17A, IP-10, and IL-8 significantly elevated in MSM-RAI; all *p* < 0.05 for individual cytokines, Supplementary Table 1). There were no significant differences between MSM-RAI and controls at days 2 (global *p* = 0.2) or 5 (global *p* = 0.6).

The twelve-cytokine inflammation score (12C IS) across all visits was significantly higher (*p* = 0.03) in the MSM-RAI group when compared to controls overall (Fig. 1B). LDM assessing MSM-RAI versus control differences at each visit demonstrated a significant 12C IS elevation in the MSM-RAI group at day 0 (*p* = 0.04) but no significant differences at days 2 (*p* = 0.2), 5 (*p* = 0.1), or 8 (*p* = 0.09) (see Supplemental Table 1 for all p values).

In summary, RM cytokines and inflammation scores were significantly higher at baseline in the MSM-RAI group when compared to controls and remained high after injury. In contrast, controls demonstrated significantly lower baseline RM inflammation that acutely increased after RM injury before trending back to a low baseline as the injury healed.

### Rectal mucosal pro-inflammatory cytokine concentrations increased after injury in both overlapping and distinct ways in MSM engaging in RAI and controls

The LDM comparing mean cytokine concentrations from baseline to day 2 demonstrated a significant difference (*p* = 0.02), and IL-6 (*p* = 0.0004) and IP-10 (*p* = 0.02) increased significantly between baseline and day 2 in both groups. There was no statistical interaction (*p* = 1), indicating that the pre- and post-biopsy cytokine differences were similar between the MSM-RAI and control groups. Significant differences were not detected in cytokine concentrations by LDM between baseline and days 5 (*p* = 0.7) or 8 (*p* = 0.6) (see Supplemental Table 2 for all p values).

Although not detected by LDM, mean cytokine plots (Fig. 1A) demonstrated apparent peaks in IL-1β and IL-8 concentrations in the control but not MSM-RAI group at the first post-injury visit (day 2) as well as a peak in IL-6 concentration at day 5. Thus, these specific cytokines and time-points were compared using the Mann-Whitney test in exploratory analyses. Among controls, IL-1β (*p* = 0.03) and IL-8 (*p* = 0.02) concentrations increased significantly between baseline and day 2, and IL-6 (*p* = 0.05) concentrations increased significantly between baseline and day 5. There were no significant differences in the MSM-RAI group for these specific cytokines between these time points.

### After experimentally induced rectal mucosal injury, there was a trend towards faster healing among MSM engaging in RAI compared to controls

**Fig. 2:**
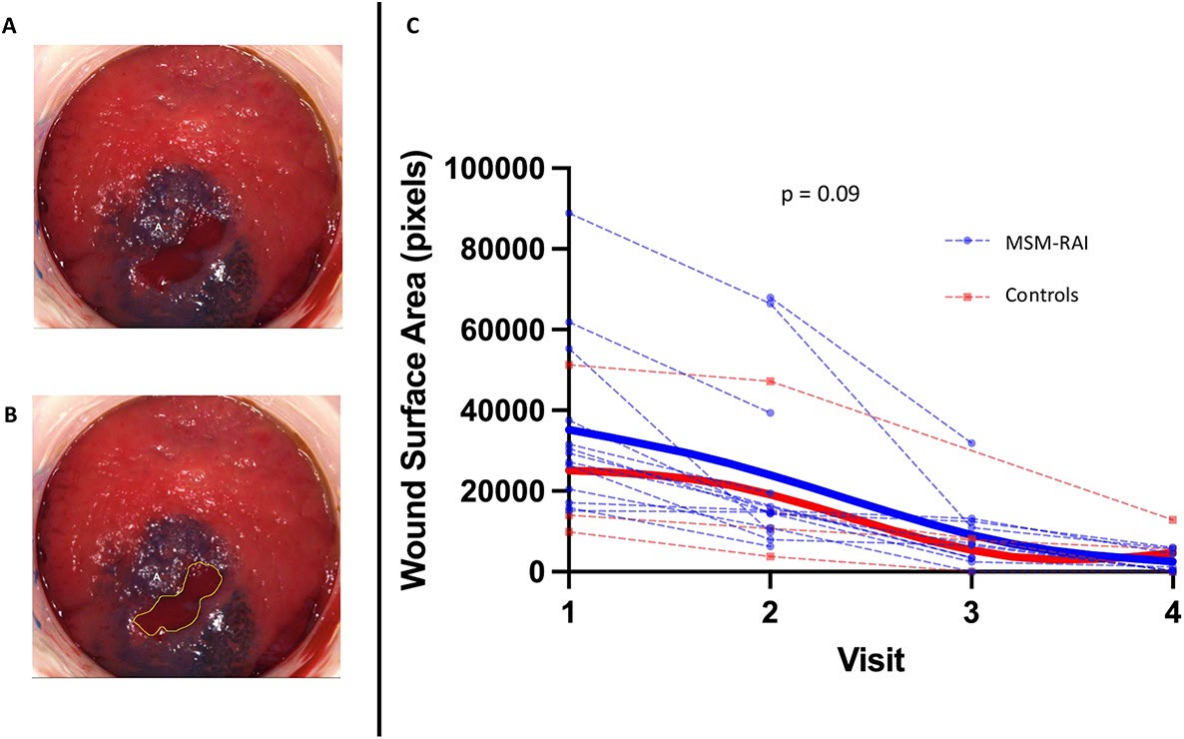
Rectal mucosal healing time after experimentally induced injury among MSM engaging in RAI and controls. (**A**) Images of experimentally induced rectal mucosal injuries were collected at baseline (day 0) and at days 2, 5, and 8, and (**B**) surface area in pixels was measured using ImageJ by outlining the wound boundary in yellow. Surface area over time was compared between MSM who have frequent RAI (blue dots) and control men who have never had RAI (red squares) using a linear mixed effects model. (**C**) depicts wound healing trajectories for all participants. The MSM-RAI and control splines are represented by thick lines.

Images of experimentally induced RM injuries were collected at baseline (day 0) and days 2, 5, and 8 (Fig. 2A). Wound surface area in pixels was measured using ImageJ (Fig. 2B**)**. Surface area over time was compared between the MSM-RAI and control groups using a linear mixed effects model. Wound healing was numerically faster in the MSM-RAI group compared to the control group, though this did not meet statistical significance (*p* = 0.09) (Fig. 2C).

### Mucosal injury was not associated with changes in the rectal mucosal microbiome composition

**Fig. 3:**
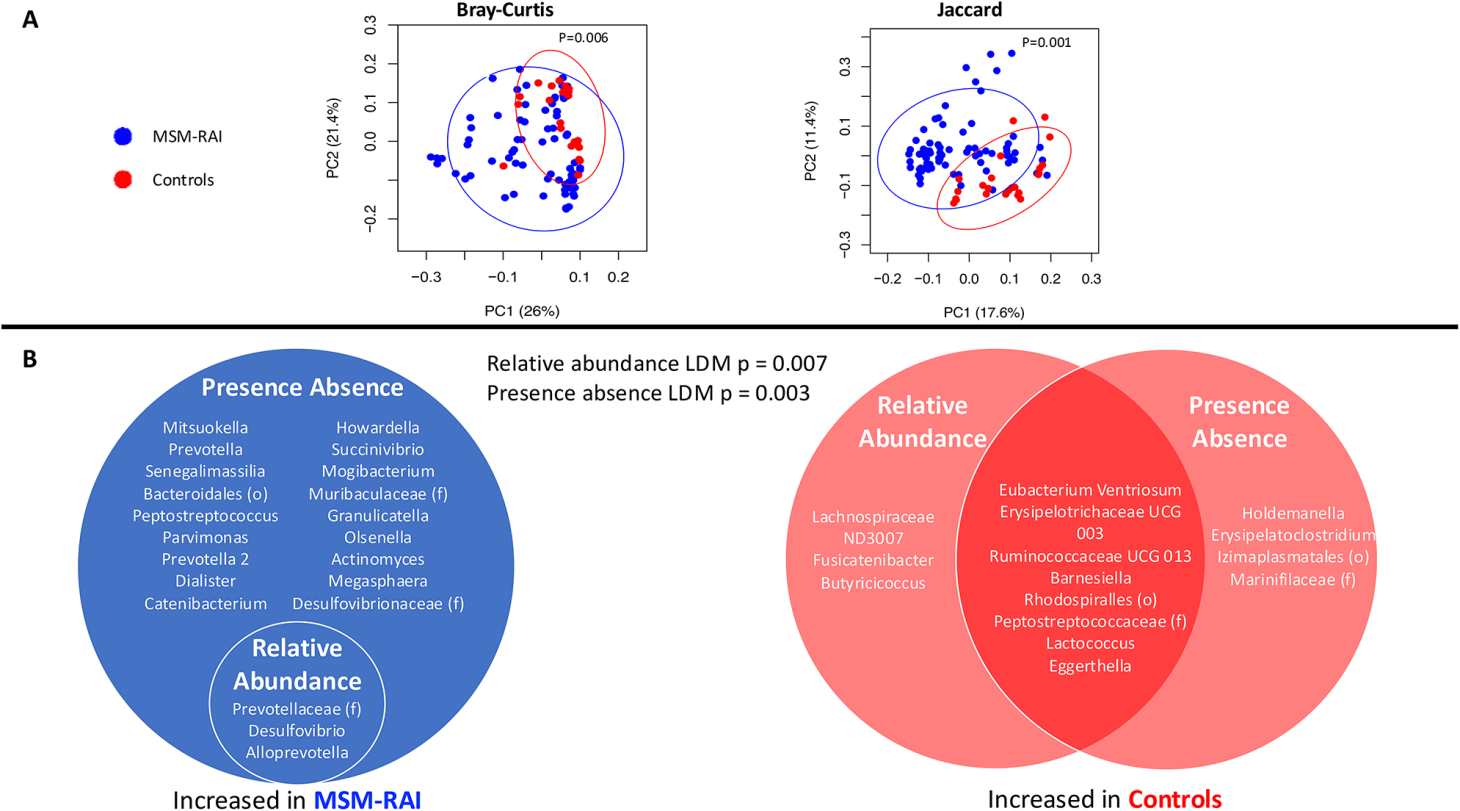
The composition of the rectal mucosal microbiome among MSM engaging in RAI and controls. Rectal microbiome composition was measured at baseline (day 0) and after experimental rectal mucosal injury (days 2, 5, and 8). (**A**) Bray-Curtis and Jaccard measures of beta diversity, measured by PERMANOVA and visualized with PCoA plots, both demonstrated significant differences between the overall microbiome composition of MSM-RAI (blue dots) and controls (red dots). (**B**) LDMs assessing relative abundance and probability of presence both demonstrated highly significant (relative abundance *p* = 0.007; presence-absence *p* = 0.003) differences between MSM-RAI and controls. The taxa listed were significant (FDR < 20%) contributors to these differences. Taxa were sequenced to the genus level when possible; those sequenced to the family (f) or order (o) level are indicated.

As has been shown previously, the rectal microbiome among MSM-RAI was distinct from controls as demonstrated by beta diversity metrics Bray-Curtis (*p* = 0.006) and Jaccard (*p* = 0.001) (Fig. 3A). LDM assessing relative abundance and presence-absence both demonstrated significant global differences (*p* = 0.007 and *p* = 0.003 respectively) between the MSM-RAI and control groups (Fig. 3B). Fifteen taxa in the relative abundance LDM and 33 in the presence-absence LDM demonstrated significant differences (FDR < 20%; see Supplemental Tables 3&4 for all p values) between MSM-RAI and controls with significant overlap in taxa represented from prior analyses^18,32,33^. There were no significant differences in alpha diversity detected between MSM-RAI and controls. There were also no significant differences in alpha or beta diversity, relative abundance, or probability of presence before or after experimentally induced injury in global or taxa-level analyses.

### The microbiome composition is associated with rectal mucosal inflammation, and distinct inflammation-associated bacterial guilds were identified in the rectal mucosa of MSM engaging in RAI and controls

**Fig. 4:**
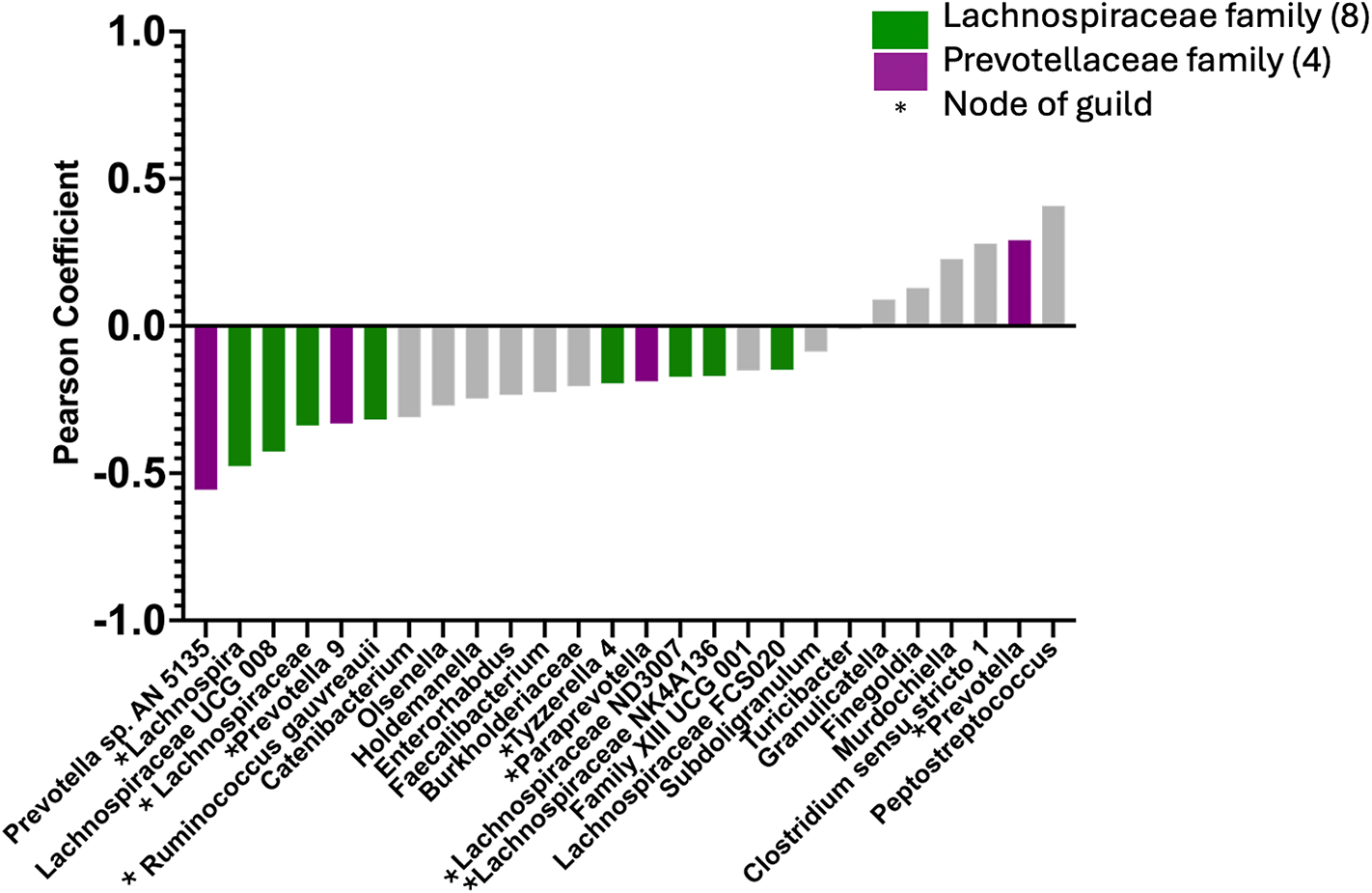
Associations between the microbiome composition and inflammation as measured by 12 cytokine inflammation score (12C IS). Pearson scores were calculated for each of the 26 taxa that were significantly correlated (FDR < 20%) with the 12C IS. The twenty taxa on the left side of the x-axis had a negative association between RA and 12 C IS. The six taxa on the right side had a positive association between RA and 12 C IS. BOUTH analysis at all available phylogenetic levels identified the *Lachnospiraceae* and *Prevotellaceae* families (*p* < 0.001 for both) as the strongest contributors to the relative abundance-12C IS LDM enrichment signal. Twelve of the 26 inflammation-associated taxa were in the *Lachnospiraceae* family (eight taxa, in green) or *Prevotellaceae* family (four taxa, in purple). SparCC network analysis identified nine of these 12 taxa as nodes of inflammation-associated guilds (*).

LDM analyses demonstrated a highly significant global association (*p* = 0.007) between the microbiome composition by relative abundance and the 12C IS. Twenty-six individual taxa were identified as significant contributors to this association (FDR < 20%; see Supplemental Table 5 for all p values). Pearson coefficients were calculated to quantify the direction and strength of associations between each of these 26 taxa and the 12C IS. Twenty taxa demonstrated a negative correlation between relative abundance and 12C IS, and six taxa demonstrated a positive correlation between relative abundance and 12 C IS. 16S rRNA sequencing generally allows resolution to the genus level. We then assessed what taxa across all phylogenetic levels were the most significant contributors to the association between relative abundance and 12C IS with BOUTH analysis. This identified two bacterial families, *Prevotellaceae* (*p* < 0.001) and *Lachnospiraceae* (*p* < 0.001), as the most significant driver taxa for the association between microbiome relative abundance and 12C IS. Twelve of the 26 taxa that were significant in the relative abundance-12 C IS LDM were members of one of these families (eight in the *Lachnospiraceae* family and four in the *Prevotellaceae* family) (Fig. 4).

**Fig. 5:**
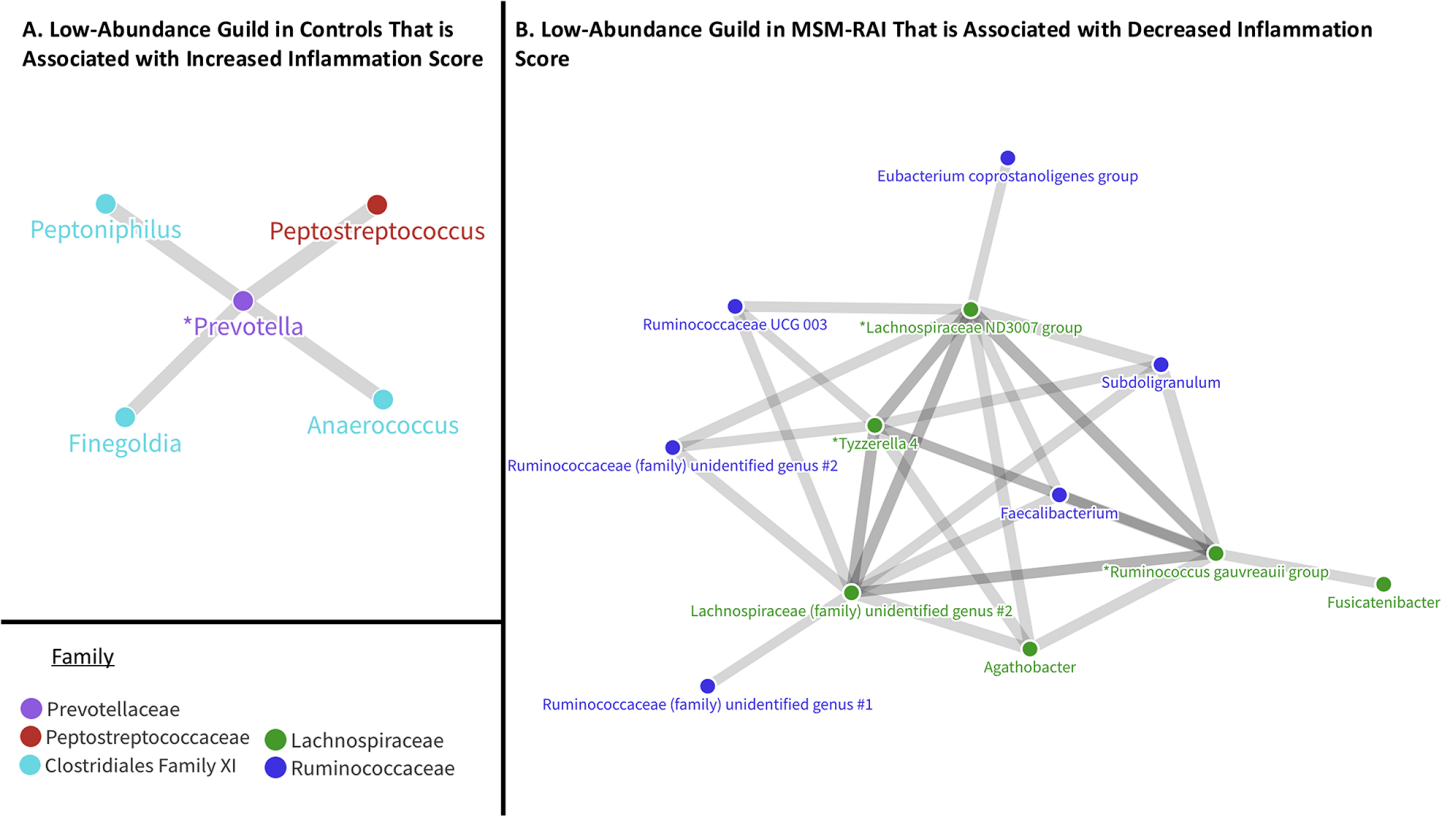
Inflammation-associated bacterial guilds among controls and MSM engaging in RAI. Flourish plots were created to visualize representative inflammation-associated guilds by mapping SparCC correlation coefficients > 0.7, represented by thick lines, between the inflammation associated nodes (*) and other bacterial taxa. Circles represent bacterial taxa and are color-coded by family. (**A**) represents a 5-taxa guild that is associated with increased RM inflammation score (12C IS) and is in decreased abundance in controls. (**B**) represents a 13-taxa guild that is associated with decreased 12C IS and is in decreased abundance in MSM-RAI.

We next performed a SparCC network analysis to assess whether these 12 inflammation-associated taxa interacted with other bacteria in guilds, or functional groups that thrive or decline together, as measured by positively or negatively correlated relative abundance without regard to taxonomic positions^29^. This analysis included 106 taxa with a relative abundance of > 0.1% and then assigned Pearson’s correlation coefficients to each pair of taxa. Of the previously identified twelve taxa of interest that were members of the *Lachnospiraceae* and *Prevotellaceae* families and significant in the relative abundance-12C IS LDM, nine were also represented in the SparCC network analysis and thus represented potential nodes of guilds associated with inflammation. We applied a stringent correlation coefficient cutoff of > 0.7 or <-0.7 to the correlations between these nine “node” taxa and the remaining 97 taxa in the SparCC analysis to identify the strongest inflammation-associated correlations. In the MSM-RAI group, twenty taxa met these criteria. When these taxa were plotted via Flourish network graphing, they formed three distinct clusters, or guilds. One guild, which was in decreased abundance in the MSM-RAI group, included 13 taxa comprised entirely of members of the *Lachnospiraceae* and *Ruminococcaceae* families. In the control group, twelve taxa met the > 0.7/<-0.7 correlation coefficient cutoff criteria. Flourish network graphing demonstrated four distinct guilds. One of these guilds, which was in decreased abundance in the control group, included five taxa comprised of anaerobic bacteria previously associated with increased inflammation, HIV target cell recruitment, and HIV acquisition^34,35^ (Fig. 5).

## Discussion

Here, we have characterized the mucosal inflammatory response to injury among MSM engaging in RAI and controls, and we have identified networks of inflammation-associated bacteria that differ between MSM-RAI and control groups. Cytokine responses differed between the two study groups, with the MSM-RAI group demonstrating overall higher IL-1β, IL-17A, IP-10, IL-8, and IFN-γ concentrations and composite inflammation scores both before mucosal injury and at the end of the healing trajectory. In contrast, inflammation was lower among controls at baseline, increased during the injury response, and then returned to a low baseline. Our data identified IL-6 and IP-10 as acute mucosal injury response mediators in both study groups. Among controls, IL-1β and IL–8 also appeared to be important injury response mediators. Our study also adds to our understanding of the crosstalk between the microbiome and mucosal immune system by identifying distinct inflammation-associated bacterial guilds in both MSM-RAI and controls.

RM inflammatory cytokine concentrations within secretions collected from the MSM-RAI group were significantly higher at baseline and remained higher than controls after experimentally induced injury. This could be attributed to physical stimulation, product use during RAI or to other unknown causes. The specific cytokines that were elevated in the RM of the MSM-RAI group, IL-8, IL-1β, IL-17A, IP-10, and IFN-γ, have known important and overlapping roles in mucosal injury and repair. IL-8, released by pro-inflammatory macrophages along with IL-1β, recruits and activates neutrophils^36^. IL-1β is a key signaling molecule that both induces initial proinflammatory cytokine production including IL-6 and contributes to tissue repair and remodeling later in the injury response^7,37^. IL-1β also stimulates T helper 17 (Th17) cell differentiation. Th17 cells subsequently secrete IL-17A, which promotes recruitment of neutrophils and other immune cells to the injury site, regulates mucosal barrier integrity and permeability, and contributes to tissue repair. Prior work from our group demonstrated that rectal CD4 + T cells from MSM engaging in frequent RAI demonstrated increased IL-17 production; it is possible that this is mediated by elevated baseline IL-1β concentration in the setting of frequent RAI^18^. IP-10 and IFN-γ are known to operate in a positive feedback mechanism to mediate wound repair by inducing chemotaxis and activation of macrophages, dendritic cells, NK cells, and T lymphocytes^38,39^. Vaginal and penile mucosal inflammation has previously been associated with HIV transmission^13,15,40,41^, and prior research evaluating highly HIV-1 exposed, seronegative MSM demonstrated that high RM innate reactivity has been associated with higher HIV acquisition risk^42^. Thus, the elevated baseline RM inflammation seen in our MSM-RAI group may have implications for HIV transmission risk in this population.

In contrast to the above cytokines that appear to be chronically elevated in the RM of the MSM-RAI group as compared to controls, IL-6 and IP-10 increased significantly in both groups after injury, underscoring their role as acute RM injury response mediators^43,44^ regardless of baseline inflammation. IL-6 has an important role in post-injury intestinal regeneration and inflammation responses and may impact microbiome composition^20,21^. As discussed above, IP-10 is a potent chemotactic agent that activates a variety of innate and adaptive immune cells at sites of mucosal injury. Of note, IL-1β and IL-8 concentrations, which are both released by pro-inflammatory macrophages during early healing as discussed above, acutely increased after injury in the control group only, suggesting that these cytokines likely mediate an acute inflammatory response in a low-inflammation environment as seen in controls but may not significantly respond in an environment of high baseline inflammation as seen in the MSM-RAI group. As previously discussed, RAI could lead to ongoing subclinical RM injury and proinflammatory cytokine production such that the experimental RM injury induced in this study had minimal additional impact on mucosal inflammation. The chronic elevation of IL-1β and IL-8 seen in MSM-RAI could trigger persistent recruitment of immune cells, including CD4+ T lymphocytes and other HIV target cells, to the RM and thus increase HIV transmission risk^45^.

We observed faster wound healing time in the MSM-RAI group that did not reach statistical significance. If wound healing is faster in MSM-RAI, it is possible that the heightened levels of RM inflammation seen in the MSM-RAI group at baseline may ‘prime’ the RM for faster injury repair as shown in a prior study comparing oral mucosal and skin transcriptional responses to injury^46^. Chronic gut inflammation has been previously linked with accelerated intestinal regeneration in patients with ulcerative colitis^47^. We hypothesize that the RM is also subjected to recurrent subclinical mucosal injury during consensual RAI similar to what has been demonstrated in the female genital tract^4^, which could prime immunologic injury and repair responses as has been seen in the female genital tract^48,49^. Therefore, the experimental mucosal injuries utilized in our study may not have generated a significant acute inflammatory response in the RM of the MSM-RAI group, whereas in the control group, experimental wound induction was a novel injury that resulted in an acute inflammatory response.

Similar to prior studies including our own, the RM microbiota among MSM-RAI demonstrated a distinct composition characterized by both higher relative abundance and probability of presence of taxa within the *Prevotellaceae* family and *Desulfovibrio* and *Alloprevotella* genera^18,32,33^. *Desulfovibrio* is the most predominant genera of sulfate-reducing bacteria in the gut and is associated with inflammation and intestinal permeability^50^. The RM microbiota among MSM-RAI also demonstrated increased probability of presence of *Prevotella* and *Succinovibrio* consistent with prior studies^32^. A recent study that assessed associations between the gut microbiome, microbial translocation, inflammation, and biological aging^51^ also found an association between increased inflammation and relative abundance of *Prevotella 2* and *Alloprevotella*. These are two of the taxa noted to be in increased probability of presence and abundance, respectively, in our MSM-RAI group. In contrast, the controls’ microbiome was characterized by increased abundance of several short chain fatty acid (SCFA)-producing taxa associated with reduced mucosal inflammation, including *Lachnospiraceae ND 3007*,* Faecalibacterium*,* Eubacterium Ventriosum*,* Ruminococcaceae UCG 013*,* Fusicatenibacter*,* Butyricicoccus*,* Erysipelotrichaceae UCG 003*,* Lactococcus*, and *Barnesiella*^52^. We describe a similar overall association in our study; in BOUTH analysis, the association between the microbiome and the 12C IS was attributed to the *Prevotellaceae* and *Lachnospiraceae* families. *Erysipelotrichaceae UCG 003*, *Peptostreptococcaceae*, and *Faecalibacterium*, all of which were in high relative abundance in controls, were also associated with decreased inflammation in a recent study^51^. Taken together, these data support an important link between mucosal inflammation and the microbiome composition MSM-RAI.

While significant microbiome alterations after mucosal injury were not observed in either study group, the analysis of inflammation-associated bacterial guilds presented here adds important nuance to our understanding of the microbiome and mucosal immune system relationship by identifying groups of co-abundant bacteria in the setting of RM inflammation. In the RM of MSM-RAI, the largest guild was comprised of 13 positively correlated *Lachnospiraceae* and *Ruminococcaceae* family members, which include some of the most significant butyrate-producing bacteria associated with inflammation attenuation^53^. *Lachnospiraceae ND3007 group* and *Fusicatenibacter* both had significantly lower relative abundance in MSM-RAI in our study, and all 13 taxa demonstrate strong positive associations with their correlated taxa, indicating that this guild is likely to be in low abundance collectively in MSM-RAI. Relative abundances of the two inflammation-associated nodes of this guild, *Lachnospiraceae ND3007 group* and *Tyzzerella 4*, as well as *Ruminococcus gauvreauii group*,* Faecalibacterium*,* Fusicatenibacter*, and *Subdoligranulum* were all negatively correlated with 12C IS, meaning that when abundance of these taxa is low, inflammation tends to be higher. *Ruminococcus gauvreauii group*,* Faecalibacterium*, and *Subdoligranulum* were also associated with decreased inflammation in a recent study^51^.

*Prevotella*, which was one of the nodes that was positively correlated with inflammation score in our analysis, had decreased abundance in controls when compared to MSM-RAI consistent with prior research^32,33^. In our study, it was highly positively correlated with *Peptostreptococcus* as well as three members of the *Clostridiales Family XI: Anaerococcus*,* Finegoldia*, and *Peptoniphilus*. Prior work from our group in young MSM without HIV demonstrated an association between *Prevotella*, *Peptoniphilus*, and *Anaerococcus* relative abundance and increased HIV viral replication in an ex-vivo rectal explant model^25^. Penile mucosal research has demonstrated that species within the *Peptostreptococcus*,* Prevotella*,* Finegoldia*, and *Peptoniphilus* genera are together associated with increased cytokine production, CD4 + T cell recruitment, and HIV acquisition^34,35^. This guild, which is in low abundance in our control group, may represent an important inflammation correlate with potential implications for HIV acquisition risk.

This study is limited by a modest sample size and lacked statistical power to assess many possible interactions; however, this is the first study to examine the RM immune response to injury in humans and adds significantly to our current understanding. We also employed robust statistical methods and a stringent correlation coefficient cutoff in our guild analysis, which may obscure some associations. We did not control for other factors, like diet, that may influence the gut microbiome, however our findings are consistent with prior studies of the microbiome among MSM engaging in RAI as compared to men who do not. PrEP use and the biosocial effects of race/racism are potential confounders. There is some evidence that these factors may be associated with gut microbiome alterations, although the taxa found to be differentially abundant in the setting of PrEP use or by racial category in other studies are inconsistent and largely did not overlap with the differences we found when comparing the rectal microbiome in our MSM-RAI and control groups. We utilized 16S rRNA microbiome sequencing, which is generally unable to distinguish taxa at the species or strain levels, and a two-dimensional wound healing measurement, which is unable to assess the depth or integrity of mucosal healing. Future, larger studies will be needed to resolve some of these limitations, and we feel that our findings justify such studies.

## Conclusions

To our knowledge, this work is the first experimental study of the impact of RM injury on the immune environment in healthy humans utilizing a novel method to measure RM healing by incorporating digital imaging analysis with high resolution anoscopy. We deepened our understanding of how the RM immune environment and injury response differ in men who engage in frequent RAI and experience the highest vulnerability to sexual HIV acquisition. Our findings also provide a basis for future multi-omic studies incorporating metagenomic sequencing and metabolomics to specifically identify important bacterial contributors to RM inflammation and their metabolic products to probe RM inflammation mechanisms. It is essential to understand how real-life behaviors like RAI affect the RM immune environment in order to ensure that HIV prevention measures, including a vaccine, are developed in an equitable and inclusive way.

## Electronic supplementary material

Below is the link to the electronic supplementary material.

Supplementary Material 1

Supplementary Material 2

Supplementary Material 3

Supplementary Material 4

## Data Availability

The microbiome dataset generated and analyzed during the current study is available in the NCBI repository (https://dataview.ncbi.nlm.nih.gov/object/PRJNA1133685?reviewer=d3l65k69trthc4nvvo2vd2beqr). The cytokine concentrations, 12C IS, and wound surface area data is included as a supplemental file (Supplemental File – Cytokine and Wound Healing Data).
