# Supplementary material for: Rectal mucosal inflammation, microbiome, and wound healing in men who have sex with men who engage in receptive anal intercourse
Source: Sci Rep. 2024 Dec 30;14:31598. doi: 10.1038/s41598-024-80074-1 (PMC11685717; doi:10.1038/s41598-024-80074-1)
Supplement: Supplementary file 1 — Supplementary Material 1 [file 41598_2024_80074_MOESM1_ESM.docx]

**Cytokine quantification**

Sigma Luminex cytokine extraction protocol

The thawed sponges were transferred onto 0.2 uM Spin-X centrifuge tube filters, and 300 uL of ice-cold Elution Buffer (0.25 M NaCl and 10% fetal calf serum in PBS) was added to each sponge and allowed to diffuse for 15 min at 4°C. The tubes were then centrifuged at 12,000 rpm x 20 min at 4°C. The upper filter chambers were discarded, and the eluent in the lower chamber was stored at –80°C.

LegendPlex Multi-Analyte Flow Assay Kit

We used the 13-plex Human Essential Immune Panel, which measures IL-4, IL-2, IP-10, IL-1β, TNF-α, MCP-1, IL-17A, IL-6, IL-10, IFN-γ, IL-12p70, IL-8, and free active TGF-β1. A 96-well plate was prepared with eight standard wells and 40 sample wells in duplicate according to manufacturer protocol. Each well was prepared with 25 ul extracted rectal mucosal cytokines or standard, 25 ul pre-mixed antibody-immobilized beads, and 25 ul assay buffer, sealed with foil, and shaken at 800 rpm for two hours at room temperature to bind the 13 cytokines to the beads. The plate was then centrifuged, the supernatant was removed, the bound cytokines were serially washed, 25 ul detection antibody was added, and the plate was again sealed in foil and incubated at room temperature for an hour. 25 ul Streptavidin PE was then added to each well, and the plate was covered in foil and incubated for 30 minutes. The plate was again centrifuged and serially washed. Each well was resuspended in 150 uL wash buffer and transferred to FACS tubes, and the cytokine concentrations were measured by flow cytometry. R2s for all standard curves were >0.99. The two duplicate concentrations for each sample were averaged. Concentrations below the limit of detection were replaced with a value of ½ of the limit of detection. Ninety-five percent (93/98) of the IL-2 concentrations were below the limit of detection, and so this data was not included in subsequent analyses.

12C IS inflammation score calculation

For each visit, participants received +1 point for each proinflammatory cytokine (IP-10, IL-1β, TNF-α, MCP-1, IL-17A, IL-6, IFN-γ, IL-12p70, IL-8) that was in the top quartile concentration and –1 point for each anti-inflammatory cytokine (IL-4, IL-10, TGF-β1) that was in the top quartile concentration for a maximum score of 9 and minimum score of –3.

**Microbiota sequencing**

DNA was extracted using the Qiagen DNeasy PowerSoil Pro Kit. Libraries were made using a modification of the Illumina 16S Metagenomic Sequencing Library Preparation workflow. 12.5 ng of DNA was amplified using 16S Amplicon PCR Forward and Reverse Primers. Libraries were purified with Ampure XP beads. Purified amplicons were indexed with Nextera XT Index primers, and indexed amplicons were purified with Ampure XP beads. Final 16S libraries were approximately 630 bp and were pooled in equal amounts based on fluorescence quantification. Final library pools were quantitated via qPCR. The pooled library was sequenced on an Illumina MiSeq using MiSeq v3 600 cycle chemistry at a loading density of 6–8 pM with 20% PhiX, generating roughly 20 million, 300 bp paired-end reads. Recommended sequencing depth was >100,000 reads per sample. Raw amplicon sequence reads were evaluated for quality control (QC) using the FastQC suite with MultiQC(1, 2) and were then processed using Quantitative Insights into Microbial Ecology (QIIME2 v2021.2)(3). The Divisive Amplicon Denoising Algorithm 2 (DADA2) package(4) was used within QIIME2 to denoise and dereplicate all paired-end sequences and to create the feature table of amplicon sequence variants used within QIIME2. DADA2 parameters were chosen to trim the first 30 bp and to truncate both paired-end reads at position 240. Taxonomic assignment was performed via QIIME2 and the data were aligned to Silva (v132)(5) using the QIIME taxonomy modules.

**Statistical Methods**

Linear decomposition modeling (LDM)

LDM allows inclusion of multiple study groups and visits into a single model, controls for multiple comparisons, and is a single analysis pathway that combines both global test of effect as well as tests of individual outcomes(8, 9). The LDM essentially fit a linear model for the cytokine concentration, inflammation score, taxon relative abundance, or taxon presence absence data as the outcome and regressed it on continuous traits or categorical group variables while adjusting for potential confounders; however, it differs from standard linear regression in that it uses permutation-based p values to account for non-normally distributed data. For these analyses, data were normalized to have mean zero and standard deviance of 1; missing data were imputed by the mean of the observed data.

Presence-absence analysis

Presence-absence analysis is the preferred analysis for identifying rare taxa. Many associations are driven by changes in which taxa are present and which are absent, however confounding by read depth is a limitation to this analysis. Our method of evaluating presence-absence involves first rarifying the ASV table such that all samples have the same library size, which eliminates confounding by read depth, and then repeatedly applying the LDM to all (i.e., infinitely) rarified taxa count tables(9).

References

1. Andrews S. Fast QC: a quality control tool for high throughput sequence data 2010 [Available from: <http://www.bioinformatics.babraham.ac.uk/projects/fastqc>.

2. Ewels P, Magnusson M, Lundin S, Käller M. MultiQC: summarize analysis results for multiple tools and samples in a single report. Bioinformatics. 2016;32(19):3047-8.

3. Bolyen E, Rideout JR, Dillon MR, Bokulich NA, Abnet CC, Al-Ghalith GA, et al. Reproducible, interactive, scalable and extensible microbiome data science using QIIME 2. Nat Biotechnol. 2019;37(8):852-7.

4. Callahan BJ, McMurdie PJ, Rosen MJ, Han AW, Johnson AJ, Holmes SP. DADA2: High-resolution sample inference from Illumina amplicon data. Nat Methods. 2016;13(7):581-3.

5. Quast C, Pruesse E, Yilmaz P, Gerken J, Schweer T, Yarza P, et al. The SILVA ribosomal RNA gene database project: improved data processing and web-based tools. Nucleic Acids Res. 2013;41(Database issue):D590-6.

6. Van Doren VE, Smith SA, Hu YJ, Tharp G, Bosinger S, Ackerley CG, et al. HIV, asymptomatic STI, and the rectal mucosal immune environment among young men who have sex with men. PLoS Pathog. 2023;19(5):e1011219.

7. Ackerley CG, Smith SA, Murray PM, Amancha PK, Arthur RA, Zhu Z, et al. The rectal mucosal immune environment and HIV susceptibility among young men who have sex with men. Frontiers in Immunology. 2022;13.

8. Hu YJ, Satten GA. Testing hypotheses about the microbiome using the linear decomposition model (LDM). Bioinformatics. 2020;36(14):4106-15.

9. Hu YJ, Lane A, Satten GA. A rarefaction-based extension of the LDM for testing presence-absence associations in the microbiome. Bioinformatics. 2021.
