# Supplementary material for: Rectal mucosal inflammation, microbiome, and wound healing in men who have sex with men who engage in receptive anal intercourse
Source: Sci Rep. 2024 Dec 30;14:31598. doi: 10.1038/s41598-024-80074-1 (PMC11685717; doi:10.1038/s41598-024-80074-1)
Supplement: Supplementary file 2 — Supplementary Material 2 [file 41598_2024_80074_MOESM2_ESM.docx]

Supplemental Tables

|  | Overall  (LDM p = 0.09) | Day 0  (LDM **p = 0.05**) | Day 8  (LDM p = 0.09) |
| --- | --- | --- | --- |
| IL-1B p (FDR) | **0.04** (0.1) | **0.03** (0.07) | **0.02** (0.1) |
| IL-17A p (FDR) | **0.03** (0.1) | **0.03** (0.07) | **0.05** (0.1) |
| IP-10 p (FDR) | **0.02** (0.1) | **0.01** (0.06) | **0.01** (0.1) |
| IL-8 p (FDR) | **0.05** (0.1) | **0.01** (0.06) | **0.03** (0.1) |
| IFN-y p (FDR) | **0.05** (0.1) | **0.03** (0.07) | 0.2 (0.3) |
| TNF-a p (FDR) | 0.1 (0.3) | 0.1 (0.2) | 0.2 (0.3) |
| IL-12p70 p (FDR) | 0.5 (0.7) | 0.5 (0.6) | 0.8 (0.9) |
| IL-6 p (FDR) | 0.9 (0.9) | 0.2 (0.3) | 1 (1) |
| MCP-1 p (FDR) | 0.2 (0.4) | 0.4 (0.6) | 0.2 (0.3) |
| IL-4 p (FDR) | 0.8 (0.9) | 0.7 (0.8) | 0.7 (0.9) |
| IL-10 p (FDR) | 0.3 (0.5) | 0.3 (0.4) | 0.2 (0.3) |
| TGF-B1 p (FDR) | 1 (1) | 1 (1) | 0.8 (1) |
|  | Overall | Day 0 | Day 8 |
| 12C IS p (FDR) | **0.03** (0.09) | **0.04** (0.08) | 0.09 (0.1) |

**Supplemental Table 1: Mean cytokine concentration and 12C IS inflammation score p values comparing MSM-RAI and control groups.** FDR = false discovery rate. LDM = linear decomposition modeling. 12C IS = composite 12-cytokine inflammation score.

|  | Day 0 – Day 2  (LDM **p = 0.02**) | Day 0 – Day 5  (LDM p = 0.7) | Day 0 – Day 8 (LDM p = 0.6) |
| --- | --- | --- | --- |
| IL-1B p (FDR) | 0.08 (0.3) | 0.5 (1) | 0.4 (0.8) |
| IL-17A p (FDR) | 0.6 (0.6) | 1 (1) | 1 (1) |
| IP-10 p (FDR) | **0.02** (0.1) | 0.9 (1) | 0.4 (0.8) |
| IL-8 p (FDR) | 0.2 (0.4) | 0.4 (1) | 0.2 (0.8) |
| IFN-y p (FDR) | 0.1 (0.4) | 0.9 (1) | 0.3 (0.8) |
| TNF-a p (FDR) | 0.3 (0.4) | 0.5 (1) | 0.6 (0.8) |
| IL-12p70 p (FDR) | 0.7 (0.7) | 0.8 (1) | 0.5 (0.8) |
| IL-6 p (FDR) | **0.0004 (0.004)** | 0.2 (1) | 0.7 (1) |
| MCP-1 p (FDR) | 0.3 (0.4) | 0.9 (1) | 0.6 (0.8) |
| IL-4 p (FDR) | 0.4 (0.5) | 0.5 (1) | 0.4 (0.8) |
| IL-10 p (FDR) | 0.2 (0.4) | 0.8 (1) | 1 (1) |
| TGF-B1 p (FDR) | 0.2 (0.4) | 0.4 (1) | 0.9 (1) |
|  | V1 – V2 | V1 – V3 | V1 – V4 |
| 12C IS p (FDR) | 0.5 (0.5) | 0.7 (1) | 0.9 (0.9) |

**Supplemental Table 2: Mean cytokine concentration and inflammation score p values comparing all participants between visits.** FDR = false discovery rate. LDM = linear decomposition modeling. 12C IS = composite 12-cytokine inflammation score.

| Taxa | p (FDR) | MSM-RAI Direction |
| --- | --- | --- |
| Prevotellaceae (family) | 0.004 (0.1) | **↑** |
| Desulfovibrio | 0.007 (0.2) | **↑** |
| Alloprevotella | 0.01 (0.2) | **↑** |
| Eubacterium Ventriosum | 0.001 (0.1) | **↓** |
| Erysipelotrichaceae UCG 003 | 0.001 (0.1) | **↓** |
| Lachnospiraceae ND3007 | 0.001 (0.1) | **↓** |
| Fusicatenibacter | 0.003 (0.1) | **↓** |
| Butyricicoccus | 0.003 (0.1) | **↓** |
| Ruminococcaceae UCG 013 | 0.006 (0.2) | **↓** |
| Barnesiella | 0.006 (0.2) | **↓** |
| Rhodospiralles (order) | 0.008 (0.2) | **↓** |
| Peptostreptococcaceae (family) | 0.008 (0.2) | **↓** |
| Lactococcus | 0.009 (0.2) | **↓** |
| Faecalibacterim | 0.01 (0.2) | **↓** |
| Eggerthella | 0.01 (0.2) | **↓** |

**Supplemental Table 3: Taxa with significantly different microbiome relative abundance between MSM-RAI and controls.** FDR = false discovery rate. MSM-RAI = men who have sex with men engaging in receptive anal intercourse.

| Taxa | p (FDR) | MSM-RAI Direction |
| --- | --- | --- |
| Desulfovibrio | 0.001 (0.08) | ↑ |
| Alloprevotella | 0.001 (0.08) | ↑ |
| Prevotellaceae (family) | 0.002 (0.08) | ↑ |
| Mitsuokella | 0.003 (0.08) | ↑ |
| Prevotella | 0.003 (0.08) | ↑ |
| Senegalimassilia | 0.004 (0.08) | ↑ |
| Bacteroidales (order) | 0.004 (0.08) | ↑ |
| Peptostreptococcus | 0.006 (0.1) | ↑ |
| Parvimonas | 0.006 (0.1) | ↑ |
| Prevotella 2 | 0.008 (0.1) | ↑ |
| Dialister | 0.009 (0.1) | ↑ |
| Catenibacterium | 0.009 (0.1) | ↑ |
| Howardella | 0.01 (0.1) | ↑ |
| Succinivibrio | 0.02 (0.2) | ↑ |
| Mogibacterium | 0.02 (0.2) | ↑ |
| Muribaculaceae (family) | 0.02 (0.2) | ↑ |
| Granulicatella | 0.02 (0.2) | ↑ |
| Olsenella | 0.02 (0.2) | ↑ |
| Actinomyces | 0.02 (0.2) | ↑ |
| Megasphaera | 0.03 (0.2) | ↑ |
| Desulfovibrionaceae (family) | 0.03 (0.2) | ↑ |
| Ruminococcaceae UCG 013 | 0.03 (0.2) | ↓ |
| Erysipelotricaceae UCG 003 | 0.001 (0.08) | ↓ |
| Holdemanella | 0.002 (0.08) | ↓ |
| Eubacterium Ventriosum | 0.004 (0.08) | ↓ |
| Rhodospiralles (order) | 0.004 (0.08) | ↓ |
| Erysipelatoclostridium | 0.007 (0.1) | ↓ |
| Lactococcus | 0.008 (0.1) | ↓ |
| Barnesiella | 0.01 (0.1) | ↓ |
| Peptostreptococcaceae (family) | 0.01 (0.1) | ↓ |
| Eggerthella | 0.02 (0.2) | ↓ |
| Izimaplasmatales (order) | 0.03 (0.2) | ↓ |
| Marinifilaceae (family) | 0.03 (0.2) | ↓ |

**Supplemental Table 4: Taxa with significantly different probability of presence between MSM-RAI and control groups.** FDR = false discovery rate. MSM-RAI = men who have sex with men engaging in receptive anal intercourse.

| Taxa | p (FDR) |
| --- | --- |
| Lachnospiraceae - uncultured | 0.0006 (0.11) |
| Ruminococcus gauvreauii group | 0.001 (0.11) |
| Lachnospira | 0.003 (0.14) |
| Burkholderiaceae (f) | 0.004 (0.14) |
| Prevotella 9 | 0.005 (0.14) |
| Lachnospiraceae ND 3007 group | 0.006 (0.14) |
| Lachnospiraceae FCS020 group | 0.007 (0.14) |
| Faecalibacterium | 0.007 (0.14) |
| Tyzzerella 4 | 0.007 (0.14) |
| Peptostreptococcus | 0.008 (0.14) |
| Prevotella sp AN 5135 | 0.008 (0.14) |
| Murdochiella | 0.01 (0.16) |
| Clostridium sensu stricto 1 | 0.01 (0.16) |
| Prevotella | 0.01 (0.16) |
| Clostridiales Family XIII UCG 001 | 0.01 (0.16) |
| Lachnospiraceae NK4A136 group | 0.01 (0.16) |
| Lachnospiraceae UCG 008 | 0.01 (0.17) |
| Olsenella | 0.02 (0.17) |
| Finegoldia | 0.02 (0.18) |
| Holdemanella | 0.02 (0.18) |
| Paraprevotella | 0.02 (0.19) |
| Subdoligranulum | 0.02 (0.19) |
| Catenibacterium | 0.02 (0.19) |
| Turicibacter | 0.02 (0.19) |
| Granulicatella | 0.02 (0.19) |
| Enterorhabdus | 0.03 (0.19) |

**Supplemental Table 5: Taxa with significant association between relative abundance and 12C IS.** FDR = false discovery rate.
