# Supplementary figures and images for: Rectal mucosal inflammation, microbiome, and wound healing in men who have sex with men who engage in receptive anal intercourse
Source: Sci Rep. 2024 Dec 30;14:31598. doi: 10.1038/s41598-024-80074-1 (PMC11685717; doi:10.1038/s41598-024-80074-1)

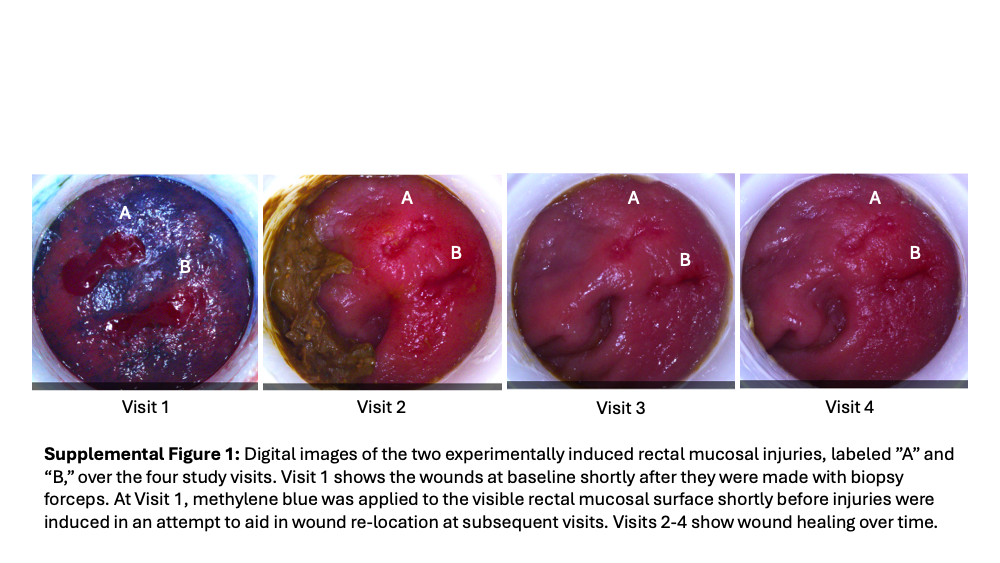

Supplement: Supplementary file 4 — Supplementary Material 4 [file 41598_2024_80074_MOESM4_ESM.tiff]
